# Complete Genome Sequences of 10 Xanthomonas oryzae pv. oryzae Bacteriophages

**DOI:** 10.1128/MRA.00334-19

**Published:** 2019-07-03

**Authors:** Tamás Kovács, János Molnár, Ildikó Varga, Ildikó K. Nagy, Sarshad Koderi Valappil, Szilvia Papp, Casiana M. Vera Cruz, Ricardo Oliva, Tímea Vizi, György Schneider, Gábor Rákhely

**Affiliations:** a Department of Biotechnology, Nanophagetherapy Center, Enviroinvest Corporation, Pécs, Hungary; b Biopesticide Ltd., Pécs, Hungary; c Department of Biotechnology, University of Szeged, Szeged, Hungary; d International Rice Research Institute, Los Baños, Philippines; e Roche Hungary Ltd., Budaörs, Hungary; f Institute of Medical Microbiology and Immunology, University of Pécs, Pécs, Hungary; g Institute of Biophysics, Biological Research Center, Szeged, Hungary; Queens College

## Abstract

Xanthomonas oryzae pv. oryzae is the causative agent of bacterial leaf blight of rice. The application of bacteriophages may provide an effective tool against this bacterium. Here, we report the complete genome sequences of 10 newly isolated OP2-like X. oryzae pv. oryzae bacteriophages.

## ANNOUNCEMENT

Bacterial leaf blight (BLB) of rice is a devastating disease causing severe economic losses, especially in Asia and western Africa (1). The etiologic agent of this infection is the Gram-negative bacterium Xanthomonas oryzae pv. oryzae (2). Due to the low efficacy of current BLB treatment tools, the emergence of resistance in X. oryzae pv. oryzae against applied agents, and public health concerns, an efficient, flexible, and environmentally sound approach is needed for controlling BLB.

The application of bacteriophages provides an alternative option for defense against plant-pathogenic bacteria, including X. oryzae pv. oryzae (3). Phages against X. oryzae pv. oryzae have been isolated extensively (4–7). Based on their morphological and serological features, Wakimoto (8) classified X. oryzae pv. oryzae phages into two major groups, OP1 and OP2. Kuo et al. isolated and characterized a morphologically distinct type of *Caudovirales* (Xp20) and a filamentous phage (Xf) (4). Recently, five new OP2-like bacteriophages were isolated and characterized, but their complete genome sequences have not been determined until now (9). The complete genomes of OP1, OP2, Xp10, and Xop411 phages have been determined (10–13); however, no complete genome sequences of other OP2-like bacteriophages have been determined until now.

For bacteriophage isolation, infected leaves originating from the Mekong Delta, Vietnam, were collected in the summer of 2014, and infected leaves, paddy water, and soil were collected from the Philippines in the summer of 2016. Either 10-g soil samples were suspended in 50 ml PSA medium (14) or 25-ml filtered water samples were mixed with 25 ml PSA medium and shaken at 160 rpm at 28°C for 24 h. Cultures were sieved through sterilized gauze and centrifuged at 2,600 × *g* for 30 min at 4°C. Supernatants were filtered using 0.2-μm syringe filters. Twenty milliliters of filtrate was supplemented with 20 ml of PSA medium, 40 μl of 1 mM MgCl_2_, 40 μl of 1 mM CaCl_2_ and 250 μl of overnight cultures of X. oryzae pv. oryzae strains LMG 641 and LMG 796 (10^8^ CFU ml^−1^). The phage lysates were filtered using 0.2-μm syringe filters and stored at 4°C until further study. The presence of lytic phages was tested by spotting 10 μl filtrate onto an X. oryzae pv. oryzae bacterial lawn grown on PSA medium and incubating further at 28°C. All isolates were purified by three successive single-plaque isolation methods using the classical drop-on-lawn technique (15).

Phage nucleic acid was isolated by using the High Pure viral nucleic acid kit (Roche Diagnostics GmbH, Germany), according to the manufacturer’s instructions. Genomic sequences of the X. oryzae pv. oryzae phages of this study were determined with MiSeq (Illumina, Inc., USA) next-generation sequencing (NGS) equipment, using Nextera XT kit (Illumina Inc.) for paired-end library preparation and Illumina V2 sequencing kit (Illumina Inc.), according to the guidelines of the manufacturer, resulting in 2,649,822 (250-bp-long) reads. The mean coverages were between 409× (XPP8) and 10,461× (XPP2).

The next-generation reads were analyzed for quality using the FastQC program (Babraham Bioinformatics, version 0.11.5), with default parameters. Low-quality bases and reads were trimmed and/or removed using the Trim Galore! (Babraham Bioinformatics, version 0.4.4 with paired mode) and Trimmomatic (version 0.36 with paired mode and using the CROP:150 MINLEN:150 parameters) programs (16). The quality-filtered reads were assembled using the MyPro software package (17). In the assembly processes, we used the Assembly.py and Integrate.py python scripts for all samples.

Genome annotation was performed using the RAST server (18), with manual curation. Each hypothetical or conserved protein-encoding gene was subjected to a search using NCBI blastx against the nonredundant protein (nr) database (19). Results were accepted when the E value was lower than e−10 and the coverage was higher than 75% as a cutoff for notable similarity.

The complete genomes of all 10 X. oryzae pv. oryzae phages were assembled. Table 1 contains the sequence lengths and G+C mol% of the newly isolated 10 phages and of the reference OP2. The G+C mol% contents of the newly isolated phages were in the range of 60.0 to 62.4 mol%, similar to that of OP2, with the exception of XPV2, for which it was higher (64.3 mol%; Table 1). The presence of 77- to 3,411-bp direct terminal repeats was detected (with a self dot plot, using the Geneious 8.0.5 software) in 6 newly isolated phages (Table 1). The complete genome nucleotide sequences of the phages were compared by pairwise alignments using the Geneious 8.0.5 software. Phage genome circularity was analyzed *in silico*. SAMtools/bcftools (20) was used to map the (raw) reads against the complete genomic sequence of the phage from whose genome sequence the reads originated. The positions of the mate-paired reads were investigated with IGV 2.5.2 (21). The circularity of the genomes was determined by observing mate-paired reads where the distance between the two reads spanned the whole genome. All of the investigated genomes were determined to be circular.

**TABLE 1:** Genome data of *X. oryzae* pv. oryzae bacteriophages

Bacteriophage	Genome length (bp)	Terminal repeat length (bp)	G+C mol%	GenBank accession no.	SRA accession no.
XPP1	46,195	0	62.4	MG944227	SAMN11254550
XPP2	46,204	276	61.0	MG944228	SAMN11254551
XPP3	46,201	3,411	62.2	MG944229	SAMN11254552
XPP4	46,200	1,197	62.1	MG944230	SAMN11254553
XPP6	46,204	77	61.0	MG944231	SAMN11254554
XPP8	46,184	0	62.2	MG944232	SAMN11254555
XPP9	46,201	2,485	61.0	MG944233	SAMN11254556
XPV1	46,503	0	60.0	MG944234	SAMN11254557
XPV2	45,969	0	64.3	MG944235	SAMN11254558
XPV3	47,046	0	60.0	MG944236	SAMN11254559
OP2	46,643	71	60.9	AP008986^*a*^	

a Sequencing of the OP2 genome was performed by Inoue et al. (10).

Genome sequencing of the newly isolated phages proved that these are OP2-like, with whole-genome nucleotide sequence similarities of 90.7 to 91.6% compared to OP2. XPV phages isolated from the Mekong Delta (Vietnam) had sequence identities of 93.2 to 96.3% compared to each other and identities of 93.0 to 94.7% compared to XPP phages isolated from the Philippines. Differences between the complete genome nucleotide sequences of the XPP phages were limited.

To the best of our knowledge, this work was the first in which the complete genomes of OP2-like X. oryzae pv. oryzae phages were determined.

### Data availability.

The complete genome sequences of newly sequenced X. oryzae pv. oryzae bacteriophages have been submitted to GenBank, and accession numbers (listed in Table 1) were assigned. The BioProject number is PRJNA529058.
